# Bedaquiline resistance-associated genetic mutations and minimum inhibitory concentrations in drug-resistant tuberculosis clinical isolates from Limpopo province, South Africa

**DOI:** 10.3389/fmicb.2026.1803115

**Published:** 2026-05-21

**Authors:** Pleasure Malope, Molebogeng Ruth Lekalakala-Mokaba, Dumisani Cyril Ngcamu, Halima Said, Ivy Rukasha, Shaheed Vally Omar

**Affiliations:** 1 Division of Medical Microbiology, Department of Pathology, Faculty of Health Sciences, University of Limpopo, Mankweng, South Africa; 2 Centre for Tuberculosis, National Institute of Communicable Diseases, Johannesburg, South Africa; 3 Department of Medical Microbiology, National Health Laboratory Services, Polokwane, South Africa

**Keywords:** bedaquiline, critical concentration, drug susceptibility testing, minimum inhibitory concentration, mutation, *Mycobacterium tuberculosis*

## Abstract

**Background:**

Bedaquiline (BDQ) resistance is a significant threat to tuberculosis (TB) control efforts, particularly in areas with a high burden of multidrug-resistant (MDR) and extensively drug-resistant (XDR) tuberculosis, such as South Africa. Determining the minimum inhibitory concentration (MIC) of BDQ and identifying the associated resistance-conferring mutations are critical for understanding resistance patterns and guiding therapeutic decisions. This study investigated BDQ resistance in *Mycobacterium tuberculosis* isolates across five districts in Limpopo, South Africa, by analyzing the relationship between minimum inhibitory concentrations (MICs) and resistance-conferring mutations.

**Method:**

This study is a cross-sectional study including 147 drug-resistant (DR-TB) isolates obtained from the Polokwane laboratory, Limpopo province, South Africa. The study used phenotypic MIC drug susceptibility testing and whole-genome sequencing (WGS) to investigate BDQ susceptibility patterns. MIC determination and genetic analysis were performed on all isolates with an MIC of >1 μg/mL and a subset of those with an MIC of <1 μg/mL as a control for sequencing.

**Results:**

Of the 147 DR-TB isolates included, 128 (87.1%) had an MIC of ≤1 μg/mL (susceptible), while 19 (12.9%) had an MIC of >1 μg/mL (resistant). Whole-genome sequencing revealed that mutations in *Rv0678* (89.1%), including frameshift and codon substitution variants, were mainly associated with drug resistance, while mutations in the *atpE* (5.1%) and *Rv1979c* (5.1%) were also identified. Of the BDQ-R strains, the Euro-American (L4) lineage was dominant, accounting for 62.5%.

**Conclusion:**

In this study, BDQ resistance was primarily driven by Rv0678 mutations. As BDQ is one of the few potent drugs available for treating DR-TB, there is a critical need for early detection and continuous surveillance to prevent the spread of resistance.

## Introduction

Tuberculosis (TB) remains the leading cause of death from a single infectious agent, with *Mycobacterium tuberculosis* responsible for approximately 1.25 million deaths globally in 2023 (WHO, 2023). This burden is aggravated by the rising incidence of drug-resistant TB (DR-TB), particularly rifampicin- and multidrug-resistant TB (RR/MDR TB) (World Health Organization, 2019). In 2023, an estimated 400,000 RR/MDR-TB cases occurred worldwide, with Africa accounting for 15.1% and South Africa contributing approximately 2.7% of the global burden (WHO, 2023; SABC NEWS, 2024).

To combat the escalating problem of DR-TB, the World Health Organization (WHO) now recommends shorter all-oral regimens that include bedaquiline (BDQ) as a core drug (WHO, 2024). BDQ plays a pivotal role in DR-TB treatment by inhibiting mycobacterial ATP synthase and is used in both short- and long-course regimens (Courbon et al., 2023). In South Africa, a high-TB burden setting, BDQ has been extensively implemented as part of standardized DR-TB treatment regimens, increasing the reliance on this drug for effective disease management. However, despite its clinical benefits, the emergence of BDQ resistance is increasingly reported, particularly in patients with prior exposure to BDQ, prolonged duration of treatment, or advanced drug resistance profiles (Ismail et al., 2022; Derendinger et al., 2023).

Genetically, resistance to BDQ has been primarily associated with mutations in the *Rv0678*, *atpE*, and *pepQ* genes. The *Rv0678* gene encodes the transcriptional repressor MmpR, which regulates the MmpS5-MmpL5 efflux pump: mutations in this gene lead to increased drug efflux and reduced susceptibility. The mutation in tis gene is generally associated with a low- to moderate-level of resistance. In contrast, mutations in the atpE gene, which encodes a transmembrane subunit of ATP synthase and the direct target of bedaquiline, are typically associated with high-level resistance. Mutations in the *pepQ* gene, a cytoplasmic peptidase, have also been implicated, although their role is less well defined (Kabahita et al., 2022; Tong et al., 2023; Chesov et al., 2022). These differences have important clinical implications, particularly for the detection of borderline resistance and treatment response.

The literature suggests that different genetic polymorphisms are associated with distinct phenotypic resistance levels (Post et al., 2004). However, the impact of these mutations on the MIC remains to be fully understood. Previous studies on genetic association for drug resistance in *M. tuberculosis* relied mainly on Drug susceptibility testing (DST)-defined phenotypes performed at a single critical concentration. However, MIC measurements provide a more accurate, precise assessment of the biological effects of genomic variation, providing a deeper understanding of resistance mechanisms (Heyckendorf et al., 2018). Correlating specific mutations associated with resistance to BDQ with MIC values is crucial to predict resistance levels and optimize treatment strategies. Understanding the link between genetic determinants and MICs can guide clinical decision-making and tailor DR-TB regimens.

Previous studies on BDQ resistance in South Africa have focused on high-burden provinces such as the Western Cape, Eastern Cape, Gauteng, and KwaZulu-Natal, where resistance rates have been extensively documented. Cape Town reported resistance rates exceeding 50% among patients with prolonged exposure to BDQ, while national surveillance data suggested an overall resistance prevalence of 2.1% in 2015 (Derendinger et al., 2023; Kaniga et al., 2022). However, data from Limpopo province are very limited due to the low level of BDQ testing in the province. Given the province’s geographical position as a border region with Mozambique, Zimbabwe, and Botswana, cross-border movement and treatment-seeking behaviors may influence unique resistance patterns that have not yet been observed in national surveillance reports (Ramaliba et al., 2017). In addition, Limpopo has a historically lower TB burden than other provinces, raising questions about whether BDQ resistance is emerging and spreading differently in the region (Hippner et al., 2019).

This study aimed to explore the relationship between MICs for BDQ and identify resistance-associated genetic mutations in *Mycobacterium tuberculosis* clinical isolates from five districts of Limpopo province, South Africa. This study is the first to investigate BDQ resistance in Limpopo using both phenotypic MIC determination and WGS.

## Methods

### Ethical consideration

Ethical approval was obtained from the Turfloop Research Ethics Committee of the University of Limpopo (TREC/564/2022: PG). The National Health Laboratory Service (NHLS) granted permission to access the isolates for research purposes through the Academic Affairs and Research Management Systems (PR2345512).

### Study site

The NHLS Pietersburg Laboratory is an academic laboratory responsible for DR-TB reflex testing in Limpopo province. It serves the entire province, which includes the following districts: Waterberg, Capricorn, Sekhukhune, Mopani, and Vhembe.

This cross-sectional study analyzed clinical isolates from patients diagnosed with pulmonary TB who attended clinics and hospitals across the five districts of Limpopo, South Africa. The study included all eligible and available isolates from the NHLS Polokwane TB repository collected between January 2021 and December 2022. Rifampicin resistance was confirmed through routine diagnostic tests, including the Xpert Ultra or the MTBDRplus line probe assay (LPA) for first-line drugs (isoniazid and rifampicin) and MTBDRsl for second-line drugs (second-line injectable agents and fluoroquinolones). Demographic information associated with the collected isolates was obtained from copies of the laboratory request forms submitted at the time of specimen submission.

### Subculture and quality control

The isolates from the repository were subcultured in the BD BACTEC MGIT™ automated mycobacterial detection system in culture medium using growth supplement and PANTA (BD, Franklin Lakes, NJ, United States) according to the manufacturer’s instructions. Once the instrument flagged positive, the isolates remained incubated at 37 °C for an additional 2 weeks to obtain more bacterial biomass. Purity testing and the presence of acid-fast bacilli (AFB) were performed using blood agar and Ziehl–Neelsen staining, respectively. A second attempt was made to recover isolates that failed to grow by resubbing from the repository vials.

### Minimum inhibitory concentration testing

The MIC of BDQ was determined using the BACTEC™ MGIT™ 960 DST method, as previously described (Torrea et al., 2015; Rüsch-Gerdes et al., 2006; Ismail et al., 2020). The EpiCenter TB-eXiST software (Becton, Dickinson and Company (BD), Franklin Lakes, New Jersey, United States) was used for the interpretation of MIC results. The standard incubation period of the MGIT 960 system was extended from 4 to 28 days to accommodate the slow-growing, drug-resistant isolates. The drug concentrations tested ranged from 0.016 to 2 μg/mL, prepared in a two-fold dilution series. All samples with MIC values above the critical concentration were tested in triplicate to further confirm the reproducibility of the MIC test. MIC results were interpreted once the drug-free growth control (GC) tube reached a growth unit (GU) value of at least 400. Each drug-containing tube was then evaluated. A sample with growth units <100 was considered sensitive, while isolates with growth units ≥100 were considered resistant at the current WHO-recommended MGIT 960 critical concentration (>1 μg/mL) (WHO, 2024). Notably, this breakpoint is considered tentative (interim) and is being further evaluated due to its small margin between wild-type and resistant strains, which may lead to discordant results. The MIC was defined as the lowest concentration of the drug-containing tube that maintained a GU of <100 at the time the GC reached a GU of ≥400. A fully drug-susceptible *M. tuberculosis* H37Rv (ATCC 27294) strain was included as a quality control in each batch; if the control failed, the entire batch was repeated.

### DNA extraction and whole-genome sequencing

DNA was extracted from MGIT cultures confirmed as *M. tuberculosis*. Briefly, a 1.2-mL aliquot of each MGIT culture was transferred to a 1.5-mL tube containing glass beads and heat-inactivated for 20 min at 80 °C in the biosafety level-3 laboratory (BSL-3). The heat-inactivated aliquots were concentrated by centrifugation at 8,000 × g for 5 min, after which 0.6 mL of the supernatant was removed. The remaining 0.6 mL was used for DNA extraction as previously described using the NucliSENS EasyMAG assay (bioMérieux, Marcyl’Étoile, France) (Ismail et al., 2023). The DNA extracts were quantified using the Qubit 4 fluorometer (Life Technologies, Carlsbad, CA, United States). Where sufficient quantified DNA was obtained, these isolates were submitted to the Sequencing Core Facility at the National Institute of Communicable Diseases (NICD) in Johannesburg, South Africa.

The WGS was performed using the NextSeq 1000 (Illumina, CA, United States). Library preparation was performed using the Illumina DNA Prep kit (Illumina, CA, United States) for paired-end sequencing, and sequencing was performed using the NextSeq reagent cartridge 300-cycle kit as per the manufacturer’s instructions. Library multiplexing was performed with an expected depth of coverage of 100× for each isolate. The quality of sequence reads was assessed using FastQC, followed by downstream bioinformatic analysis in CLC Genomics Workbench (v11.0, Qiagen, Venlo, Netherlands). Reads were mapped to the *M. tuberculosis* H37Rv reference genome (NC_000962.3) using stringent alignment parameters, with both similarity and length fractions set at 90% to ensure high-confidence mapping. Variant detection was performed using a resequencing workflow targeting mutations in the *Rv0678*, *atpE*, *Rv1979c*, and *pepQ* genes. Mutations were accepted if the quality score was ≥20 (≥99% accuracy), an average coverage depth of ≥5X, a mutation frequency of ≥10%, a minimum of 5 reads, and were present in both forward and reverse sequence reads. Resistance prediction was performed using version 1 of the WHO Catalog of Mutations (WHO, 2021).

### Data analysis

Data were captured in Excel and analyzed using version 29.0 of the Statistical Package for the Social Sciences (SPSS) software. Categorical variables were expressed as absolute numbers and percentages. Demographic data were tabulated. The results were analyzed by maintaining a laboratory log sheet to record all the MIC results. Descriptive statistics were used to determine the distribution of MICs, mutations, and the associated lineages. The Mann–Whitney U-test was used to compare MIC values between two groups, while the Kruskal–Wallis test was used for comparisons across the TB resistance profiles and lineages. Associations between categorical variables were assessed using the chi-squared test. A *p*-value < 0.05 was considered statistically significant.

## Results

**Figure 1:**
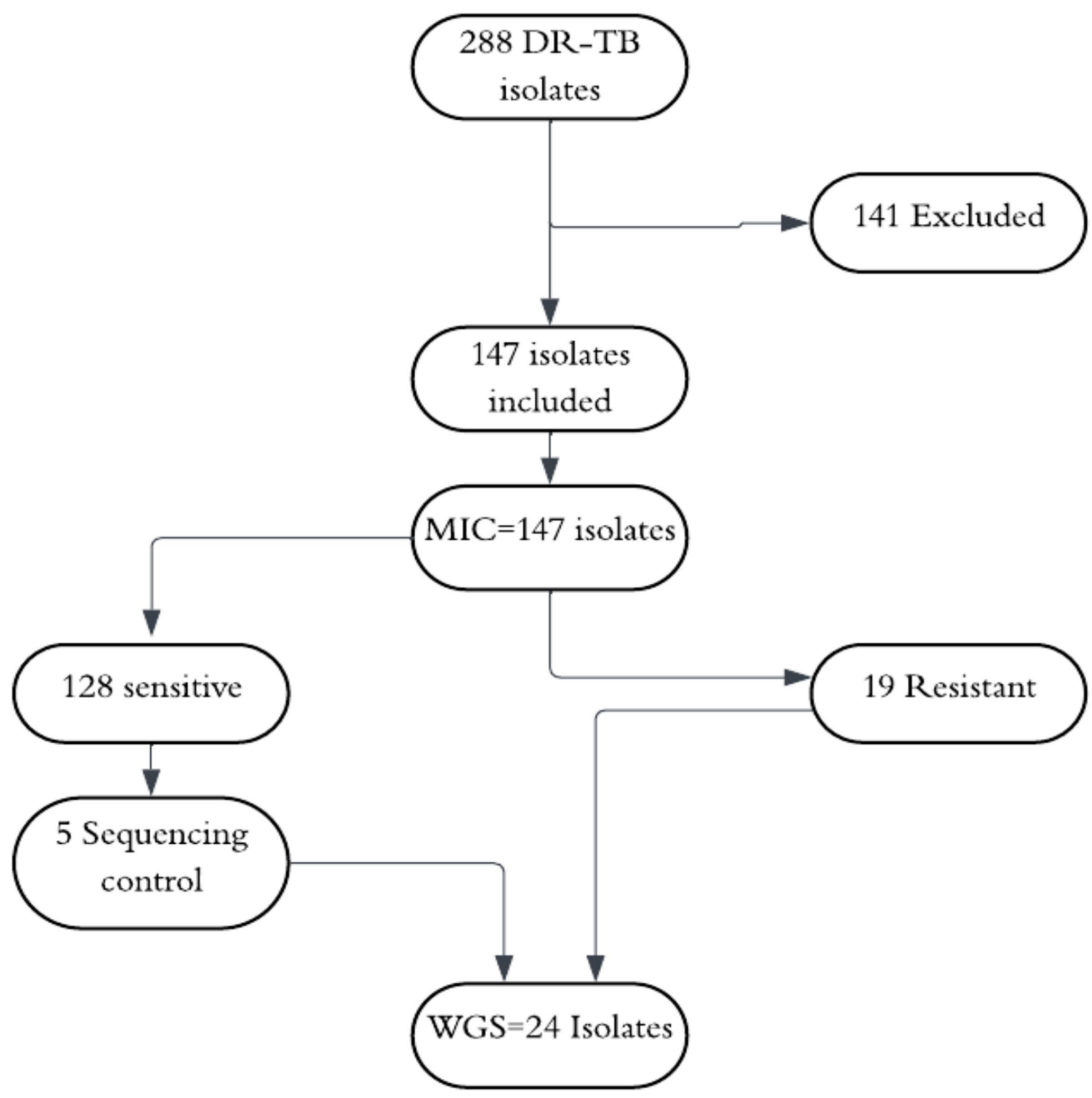
CONSORT diagram illustrates the screening and inclusion process for 288 drug-resistant tuberculosis (DR-TB) isolates from Limpopo province, South Africa.

A total of 288 isolates were prospectively collected, of which 141 were excluded from the study (90 duplicates, 7 contaminated, and 44 non-viable). Laboratory processing was completed for the remaining 147 isolates (Figure 1).

### Demographic characteristics

The majority of the isolates were from male subjects (77, 52.4%), with a mean age of 39 (IQR: 18 to 46) years. As per routine testing, 96 (65.3%) isolates were classified as rif-mono-resistant TB, 42 (25.6%) as MDR-TB, and 2 (1.4%) as XDR-TB (Table 1).

**Table 1:** Demographic characteristics and BDQ susceptibility outcomes of drug-resistant *M. tuberculosis* clinical isolates categorized by sex, age group, and TB profiles.

Characteristics		No. of clinical isolates (%)	
		BDQ^S^ (*n* = 128)	BDQ^R^ (*n* = 19)
Sex	Male	69 (90)	8 (10)
	Female	59 (84)	11 (16)
Age group	≤15	2 (100)	0 (0)
	16–30	40 (89)	5 (11)
	31–45	55 (90)	6 (10)
	46–60	21 (72)	8 (28)
	≥61	10 (100)	0 (0)
Resistance profile	Rif-mono	82 (85)	14 (15)
	MDR-TB	40 (95)	2 (5)
	Pre-XDR-TB	6 (86)	1 (14)
	XDR-TB	0 (0)	2 (100)

### Minimum inhibitory concentration test

Of the 147 isolates tested for MIC, 19 (12.9%) showed an MIC of >1 μg/mL (resistant) to BDQ. The remaining 128 isolates (87.1%) had an MIC of ≤1 μg/mL (susceptible). Among the susceptible isolates, 97 (66.0%) showed susceptibility at drug concentrations ranging from 0.125 μg/mL to 0.5 μg/mL, while 30 (20.4%) exhibited susceptibility at the borderline concentration of 1 μg/mL (Table 2).

**Table 2:** Minimum inhibitory concentrations and susceptibility breakpoints for bedaquiline (BDQ) among drug-resistant *M. tuberculosis* clinical isolates (*N* = 147).

Drug	Strain	Total number of isolates 147 (100%)	Number of isolates in the MIC								Break point (μg/m)	No (%) of resistant isolate
			0.015	0.03	0.06	0.125	0.25	0.5	1	2		
BDQ	Rif-mono	96	–	–	–	3	21	41	17	14	1	14 (15.2)
	MDR	42	–	–	–	1	10	20	8	2		2 (4.9)
	Pre-XDR	7	–	–	–	–	–	1	5	1		1 (28.6)
	XDR	2	–	–	–	–	–	–	–	2		2 (100)

### Mutation-associated drug resistance

Whole-genome sequencing was successfully performed on 24 isolates, which included 19 BDQ-resistant (MIC > 1 μg/mL) and 5 BDQ-sensitive (MIC = 1 μg/mL) isolates that were on the borderline of resistance and were used as sequencing controls. These included 14 rif-mono-resistant TB, 2 MDR-TB, 6 pre-XDR-TB, and 2 XDR-TB.

Among the 19 BDQ-resistant isolates, mutations in the *Rv*0678, *Rv*1979c, pepQ, and *atp*E genes were investigated. Mutations were observed for all genes except pepQ and are detailed in Tables 3, 4.

**Table 3:** Description of mutations associated with bedaquiline resistance isolates and their association with MICs and WGS results.

pDST	Gene	No of variant *n* (%)	Nucleotide change	Amino acid change	MIC (μg/mL)	WGS final confidence grading
R (*N* = 19)	*Rv0678*	1 (5.3)	A88T	Arg30Trp	≥2	R
		1 (5.3)	T451C	Ser151Pro	≥2	R
		2 (10.5)	G245T	Arg82Leu	≥2	R
		1 (5.3)	C265T	Arg89Trp	≥2	R
		1 (5.3)	C286T	Arg96Trp	≥2	R
		1 (5.3)	424dupC	Leu142fs	≥2	R
		1 (5.3)	144dupC	Glu49fs	≥2	R
		2 (10.5)	198dupG	Ile67fs	≥2	R
		1 (5.3)	275dup	Try92fs	≥2	R
		1 (5.3)	321dup	Ile108fs	≥2	R
		1 (5.3)	465delG	Arg156fs	≥2	R
		2 (10.5)	198delG	Ile67fs	≥2	R
		1 (5.3)	C305T	Ala102Val	2	R
		1 (5.3)	144dupC	Glu49fs	2	R
	*atpE*	1 (5.3)	C198G	Ile66Met	≥2	R
	*pepQ*	–	–	–	–	–
pre-XDR (*N* = 5)	*Rv1979c*	1 (20.0)	C1226T	Arg409Gln	1	S

**Table 4:** Supplementary table of isolates associated with bedaquiline resistance on WGS.

Patient ID	TB profile	Gene	Mutation type	Coverage	Nucleotide change	Amino acid change	Drug
OA2914	Rif-mono	*Rv0678*	SNV	77	C305T	Ala102Val	Bdq
OF4644	XDR-TB	*Rv0678*	Deletion	111	198delG	Ile67fs	Bdq
NA1080	Rif-mono	*Rv0678*	Deletion	145	465delG	Arg156fs	Bdq
		*Rv0678*	SNV	102	A88T	Arg30Trp	Bdq
PI4323	XDR-TB	*Rv0678*	Insertion	89	198dup	Ile67fs	Bdq
NB6068	Rif-mono	*Rv0678*	Insertion	88	144dup	Glu49fs	Bdq
		*Rv0678*	SNV	111	G245T	Arg82Leu	Bdq
NB8677	Rif-mono	*Rv0678*	SNV	107	G245T	Arg82Leu	Bdq
NK5548	Rif-mono	*Rv0678*	SNV	101	C265T	Arg89Trp	Bdq
		*Rv0678*	SNV	104	C286T	Arg96Trp	Bdq
NP7151	Pre-XDR-TB	*Rv0678*	Insertion	107	198dup	Ile67fs	Bdq
		*Rv0678*	Insertion	122	275dup	Tyr92fs	Bdq
NI1182	Pre-XDR-TB	*Rv1979c*	SNV	133	C1226T	Arg409Gln	Bdq
OA7902	Rif-mono	*Rv0678*	Insertion	107	321dup	Ile108fs	Bdq
		*Rv0678*	Insertion	105	424dup	Leu142fs	Bdq
OF7650	Rif-mono	*Rv0678*	SNV	97	T451C	Ser151Pro	Bdq
OS5480	Rif-mono	*Rv0678*	Insertion	92	144dup	Glu49fs	Bdq
OS8448	MDR-TB	*Rv0678*	Deletion	93	198delG	Ile67fs	Bdq
NF5136	Rif-mono	*atpE*	SNV	99	C198G	Ile66Met	Bdq

### Lineage distribution of the DR-TB isolates

**Figure 2:**
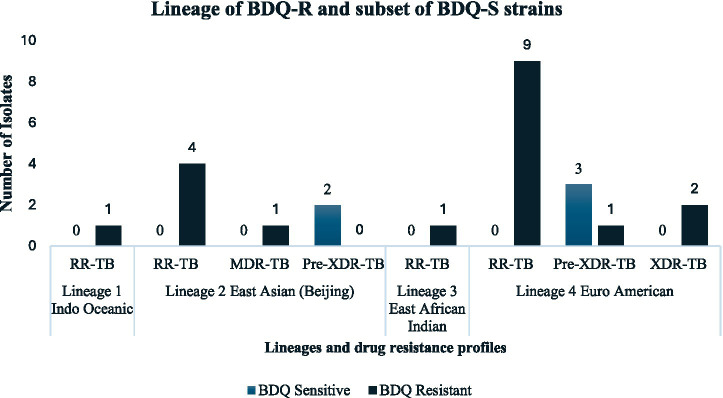
Distribution of *M. tuberculosis* lineages and their association with BDQ resistance and susceptibility among drug-resistant tuberculosis isolates from Limpopo province.

Whole-genome sequencing identified four main *M. tuberculosis* complex lineages (L): East African–Indian (L1), East Asian (including Beijing) (L2), Indo-Oceanic (L3), and Euro-American (Haarlem, H37Rv-like, LAM, and X-type) (L4). Among the 24 sequenced isolates, L4 was predominant (15/24; 62.5%), followed by L2 (7/24; 29.2%), L3 (1/24; 4.2%), and L1 (1/24; 4.2%) (Figure 2; Table 5).

**Table 5:** Statistical analysis of MIC values, resistance, mutations, and lineage among sequenced *M. tuberculosis* isolates.

Comparison	Groups	Test used	Test statistic	*p*-value	Statistical significance
MIC vs. mutation status	Mutation present vs. absent	Mann–Whitney U	*U* = 93.5	0.057	Not significant
MIC vs. TB resistance profile	RR-TB, MDR-TB, Pre-XDR-TB, pre-XDR-TB, XDR-TB	Kruskal–Wallis	*H* = 18.0	<0.001	Significant
MIC vs. lineage	Euro-American, East-African-Indian, East-Asian (Beijing), Indo-Oceanic	Kruskal–Wallis	*H* = 0.89	0.828	Not significant
Mutation vs. resistance	Mutation present vs. absent vs. R/S	Chi-square	χ^2^ = 2.09	0.149	Not significant
Lineage vs. resistance	Euro-American, East-African-Indian, East-Asian (Beijing), Indo-Oceanic vs. R/S	Chi-square	χ^2^ = 0.79	0.852	Not significant

Statistical analyses in this study showed a statistically significant difference in MIC distributions across TB resistance profiles (Kruskal–Wallis test, *p* < 0.001), indicating variability in bedaquiline susceptibility among drug-resistant TB profiles. In contrast, no statistically significant association was observed between mutation status, lineage, and phenotypic bedaquiline resistance (chi-squared test).

## Discussion

The introduction of BDQ, the first new class of anti-mycobacterial agent after 40 years, was met with great optimism. It has been associated with significantly improving patient outcomes and reducing mortality in DR-TB (Ndjeka et al., 2022). In addition to linezolid, fluoroquinolone (if susceptible), and pretomanid (BPaL), it has shortened treatment durations from 9 months for patients with DR-TB to that of DS-TB treatment of 6 months (WHO, 2022). However, these gains are at risk with the emergence of resistance to BDQ. Given this emergence, it is critical to conduct continuous population-level surveillance to understand the prevalence of drug resistance to these important agents.

In this study, BDQ resistance was observed in 12.9% of the cases. However, this finding does not represent the overall prevalence of BDQ resistance in Limpopo province, as the study used stored repository samples rather than the systematic probability-based sampling required for a true prevalence study; it nevertheless suggests a concerning level of emerging resistance, which warrants closer monitoring and targeted interventions. Resistance to BDQ was higher among pre-XDR isolates when compared to rif-mono and MDR, consistent with previous findings that the relative risk increased with fluoroquinolone-resistant isolates (Ismail et al., 2022). This could be due to cumulative drug exposure and selective pressure in patients with more complex treatment histories.

Resistance to BDQ in this study was mainly mediated by mutations in the *Rv0678 gene*, which aligns with local and global findings, confirming its central role in conferring BDQ resistance (WHO, 2022; Degiacomi et al., 2020). Although resistance to BDQ was initially expected to be due to target-based mutations in the *atp*E gene encoding subunit C of the ATP synthase complex (Chesov et al., 2022; Degiacomi et al., 2020; Omar et al., 2022; Ismail et al., 2019), non-target-based mutations in the *Rv*0678 gene encoding the MmpS5-MmpL5 efflux pump repressor were found to be dominant in our study, present in 89.5% of the clinical isolates resistant to BDQ. However, the association between these mutations and MIC values was inconsistent. The *Rv*0678 mutation has drawn significant interest due to its role in variable expression of the MmpS5-MmpL5 efflux pump, resulting in fluctuating MIC levels to BDQ, as reported in previous studies (Kaniga et al., 2022; Villellas et al., 2017). In contrast, the single *atpE* mutation identified in this study was associated with high-level resistance (MIC ≥ 2 μg/mL), supporting its role in directly altering the drug target and conferring a more consistent resistance phenotype (Karmakar et al., 2019). Another isolate carried a mutation in *Rv1979c*, which has previously been associated with low-level resistance (Saeed et al., 2022; Ismail et al., 2018). No mutations were identified in the *pepQ* gene.

Although the majority of BDQ-R isolates exhibited high MICs (MICs ≥ 2 μg/mL), some isolates with similar MICs did not show mutations on WGS. This discordance between phenotype and genotype suggests that resistance may be mediated by variants present at low frequencies within the bacterial population, falling below the detection threshold of standard WGS. This finding underscores the importance of using both phenotypic and genotypic testing to accurately assess BDQ resistance.

BDQ-R isolates in this study were predominantly found among L4 (63.2%) and L2 (31.6%) isolates. While this prevalence of BDQ resistance among L4 and L2 aligns with previous reports, these findings should be interpreted with caution, as no statistical association or phylogenetic analysis was performed in this study (Dixit et al., 2025; Atavliyeva et al., 2024; Kaapu et al., 2025). Further studies with larger sample sizes incorporating phylogenetic analysis are required to better understand the role of lineages in the epidemiology of BDQ resistance.

In this study, a notable proportion (21%) of BDQ-resistant isolates exhibited borderline resistance (MIC = 1 μg/mL). These isolates represent a clinically significant gray zone between susceptibility and resistance. Although categorized as susceptible based on current breakpoints, such isolates may harbor low-level resistance, particularly involving *Rv0678*-mediated efflux. This finding highlights the critical need for close monitoring of these isolates to prevent potential treatment failure and progression to higher resistance levels.

The observed rate of resistance to BDQ highlights the importance of universal resistance testing for all RR-TB cases in South Africa, where BDQ is a core drug for the management of DR-TB (WHO, 2022). Strengthening both phenotypic and genomic surveillance systems is essential to enable early detection of resistance-associated mutations, including those conferring borderline resistance. While conventional DST based on a single critical concentration remains the standard approach, it fails to capture the full spectrum of BDQ susceptibility observed in this study. The current WHO-recommended breakpoint for bedaquiline may require re-evaluation to better distinguish low-level resistance. Furthermore, studies on the acquisition of resistance are necessary to understand its emergence within Limpopo province. This study suggests that integrating genotypic and phenotypic data is essential for accurate resistance classification. This combined approach facilitates optimized programmatic therapeutic decisions and extends the clinical utility of BDQ in combating DR-TB.

## Limitations

This study was limited by its sample size and the reliance on a province-specific dataset, which may not be representative of the broader population. The isolates were obtained from a defined clinical repository, introducing potential sampling bias and limiting the ability to infer prevalence at the population level. Furthermore, a substantial proportion of isolates were excluded from the final analysis due to duplication, contamination, and loss of viability during subculture. These exclusions may have introduced selection bias, as the analyzed dataset may not be fully representative of the original sample population. Clinical outcome data were not available to correlate MIC and genotypic results with treatment success or failure. Additionally, socio-economic and adherence factors that may influence resistance development were not assessed. Despite these limitations, the findings provide valuable insights into the BDQ resistance landscape in Limpopo and highlight the importance of robust population-level surveillance to detect and circumvent resistance.

## Conclusion

This study identified that over 12% of RR/MDR-TB isolates submitted for BDQ phenotypic testing in Limpopo province exhibit BDQ resistance. Mutations in the *Rv0678* gene were the primary driver of resistance, while *atpE* mutations were uncommon but associated with high-level resistance. Further studies are required to understand the relationship between laboratory resistance and patient outcomes in order to understand the impact of resistance mediated by efflux.

## Data Availability

The datasets presented in this study can be found in online repositories. The names of the repository/repositories and accession number(s) can be found at: https://www.ncbi.nlm.nih.gov/, PRJNA1416012.
